# Non-cancer health risks in firefighters: a systematic review

**DOI:** 10.4178/epih.e2022109

**Published:** 2022-11-16

**Authors:** Jeong Ah Kim, Soo Yeon Song, Wonjeong Jeong, Jae Kwan Jun

**Affiliations:** 1 National Cancer Control Institute, National Cancer Center, Goyang, Korea; 2 Graduate School of Cancer Science and Policy, National Cancer Center, Goyang, Korea

**Keywords:** Occupational health, Firefighters, Musculoskeletal diseases, Post-traumatic stress disorder, Chest pain, Myocardial infarction

## Abstract

osFirefighters are occupationally exposed to hazardous factors that may increase their risk of disease. However, non-cancer disease risk in firefighters has not been systematically examined. This systematic review aimed to identify non-cancer disease risk in firefighters and determine whether the risk differs according to job characteristics. We searched the Cochrane Library, Embase, PubMed, and KoreaMed databases using relevant keywords from their inception to April 30, 2021. The Risk of Bias Assessment Tool for Non-randomized Studies version 2.0 was used to assess the quality of evidence. Due to study heterogeneity, a narrative synthesis was presented. The systematic literature search yielded 2,491 studies, of which 66 met the selection and quality criteria. We confirmed that the healthy worker effect is strong in firefighters as compared to the general population. We also identified a significant increase in the incidence of lumbar disc herniation, lower back pain, angina pectoris, acute myocardial infarction, and post-traumatic stress disorder (PTSD) in firefighters compared to other occupational groups. Contradictory results for the risk of PTSD and anxiety disorders related to rank were reported. Sufficient evidence for increased risk of lumbar disc herniation, lower back pain, angina pectoris, acute myocardial infarction, and PTSD was available. The risk of non-cancer diseases varied depending on job type, years of service, and rank. However, caution should be exercised when interpreting the results because the classification criteria for firefighters’ jobs and ranks differ by country.

## GRAPHICAL ABSTRACT

[Fig f2-epih-44-e2022109]

## INTRODUCTION

Firefighters prevent, take precautions against, or suppress fires and safeguard the lives, bodies, and properties of citizens by providing rescue and first-aid services. During fire suppression activities, firefighters are exposed to a variety of hazardous chemicals, such as smoke, particulate matter, and many other organic toxicants. Most of their working hours are on standby for shift work; thus, they are exposed to job stress. As a result, firefighters face an increased risk of respiratory, cardiovascular, mental, and cancerous disease [1,2]. Determining diseases with a higher risk of incidence and mortality in firefighters is important for prevention, intervention, and occupational safety.

Most epidemiological studies have examined cancer in firefighters compared with the general population [3-9]. Unfortunately, firefighters have a relatively high cancer incidence and mortality [10-12]. LeMasters et al. [11] reported that firefighters have a probable higher risk of multiple myeloma, non-Hodgkin lymphoma, prostate cancer, and testicular cancer. Similarly, the International Agency for Research on Cancer identified statistically significant increased risks of prostate cancer, testicular cancer, and non-Hodgkin lymphoma in firefighters [12].

However, relatively few studies have systematically reviewed non-cancer diseases. Although a systematic review of non-cancer diseases in firefighters has been previously published [13], it covered only some non-cancer diseases, such as cardiovascular disease, hearing loss (HL), mental health, respiratory illness, hip osteoarthritis, and sarcoidosis. Therefore, a more comprehensive examination of non-cancer diseases is needed. This study expanded the scope of non-cancer diseases and included studies dealing with symptoms such as pain expressed by firefighters. Hence, we systematically reviewed the literature on the incidence of non-cancer diseases in firefighters as compared to non-firefighters. Furthermore, we investigated whether the risk of noncancer diseases differs depending on job characteristics, such as job type, years of service, and rank.

## MATERIALS AND METHODS

### Study selection

This systematic review aimed to identify the risk of non-cancer diseases in firefighters. This study was conducted according to the guidelines of the Preferred Reporting Items for Systematic Review and Meta-Analyses (PRISMA) [14,15]. The inclusion criteria for participants, intervention, comparison, outcomes, and study design (PICO-SD) conformed to the specifications suggested by PRISMA [14]. The participants (P) included both male and female firefighters; the intervention (I) included firefighter work; the comparison (C) included (1) inter-comparisons (comparisons of firefighters with non-firefighters) and (2) intra-comparisons (comparisons of firefighters according to job characteristics); the outcomes (O) included non-cancer diseases; and the study design (SD) included cross-sectional, case-control, and cohort studies. Articles were excluded if they met any of the following criteria: (1) were in languages other than English or Korean; (2) evaluated simulation fire experiments or 911 terrorist incidents; (3) did not include comparisons; and (4) were not available as full-text articles.

### Search strategy

This review was performed in accordance with the PRISMA statement [14]. We searched the Cochrane Library, Embase, PubMed, and KoreaMed databases from database inception up to April 30, 2021, to identify the risk of non-cancer diseases in firefighters. Our search terms included firefighter, disease, incidence, prevalence, mortality, injury, death, and occupational level. Supplementary Material 1 presents the database search strategies.

We identified 2,491 records in the database search and excluded 876 duplicate results. The authors screened the remaining 1,615 records for eligibility and identified 93 articles eligible for full-text review. We excluded 27 studies from the full-text review because they included no comparative group, did not evaluate firefighters as a population, or were only conference abstracts. After a full-text review, 66 studies were included because they met the inclusion and exclusion criteria [16-81].

**Figure 1.**
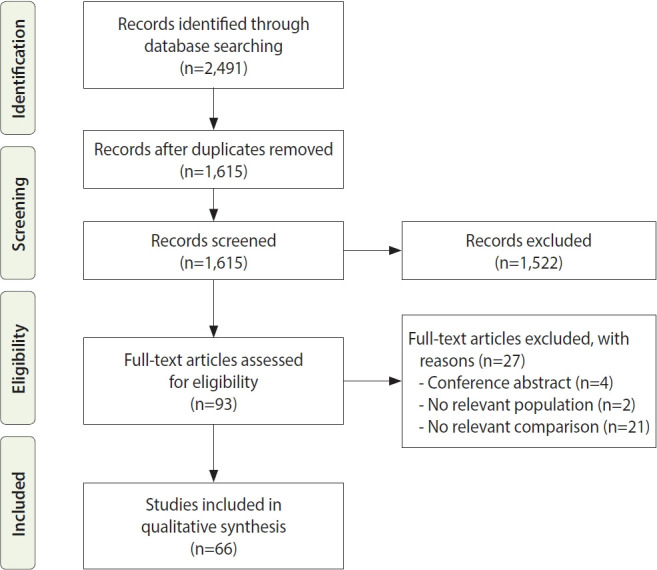
Flow diagram of study selection.

The study selection process and results at each stage are summarized in the modified PRISMA flow diagram in Figure 1 [14]. To ensure the validity and reliability of the results, two investigators independently screened the titles and abstracts of the articles and assessed the full texts for eligibility. Discrepancies were resolved through a consensus meeting with a third investigator.

### Quality assessment

Methodological quality was assessed using the Risk of Bias Assessment Tool for Non-randomized Studies (RoBANS version 2.0) [15]. RoBANS version 2.0 addresses 8 domains: (1) the possibility of target group comparisons, (2) target group selection, (3) confounders, (4) exposure measurement, (5) assessors’ bias, (6) outcome assessment, (7) incomplete outcome data, and (8) selective outcome reporting [15]. Each domain was evaluated as “low risk of bias,” “high risk of bias,” or “unclear.” Two review authors independently applied the tool to each included study and recorded supporting information and justifications for judgment of the risk of bias for each domain. Any discrepancies in judgments of risk of bias or justifications for judgments were resolved by discussion to reach a consensus between the 2 review authors, with a third review author acting as an arbiter if necessary. The results of the quality assessment were presented using Review Manager 5.4 (RevMan; Cochrane Collaboration, Oxford, UK).

### Data extraction and synthesis

One review author independently extracted all data from the eligible studies, and the characteristics of the variables were systematically organized using a coding table. The coding table included study information (author and publication year), participants (total number of participants, age, and sex), comparison group, study design, diagnostic criteria, and outcomes. This study coded studies as including male or female or both male and female firefighters in relation to sex, and no study recruited only female firefighters. The diagnostic criteria are presented in Supplementary Material 2. Our study used different measures of effect size, such as the standardized mortality ratio (SMR), standardized incidence ratio (SIR), relative risk (RR), hazard ratio (HR), odds ratio (OR), prevalence ratio (PR), incidence density ratio (IDR), and the proportionate mortality ratio (PMR), along with their 95% confidence intervals (CIs). We used the criteria for SMR, IDR, or PMR as follows: If a study reported results by multiplying the SMR, IDR, or PMR by 100, the corresponding result was divided by 100.

Due to the heterogeneity of diagnostic criteria, comparison groups, and outcomes, the findings are presented as a narrative synthesis. We created outcome categories, and overall non-cancer disease incidence and non-cancer disease mortality rates were analyzed separately. We then organized the studies according to the comparison groups. This process provided a basis for determining the studies that were eligible for each outcome.

### Ethic statement

This study was approved by the Institutional Review Board of the National Cancer Center in Korea (IRB No. NCC2021-0262).

## RESULTS

### Quality of included reviews

We used the RoBANS version 2.0 to assess the risk of bias for each of the included studies. Supplementary Material 3 provides a summary of these assessments. Overall, the risk of bias was rated as low or unclear in most identified reviews. Seventeen studies (26%) had a high risk of bias due to an improper selection of target group comparisons, 2 studies (3%) had a high risk of bias due to improper target group selection, 5 studies (8%) had a high risk of bias due to improper consideration related to confounders, 18 studies (27%) had a high risk of bias due to improper exposure measurement, 8 studies (12%) had a high risk of bias due to improper outcome assessment, and 7 studies (11%) had a high risk of bias due to improper handling of incomplete outcome data. Furthermore, in incomplete outcome data and selective outcome reporting, more studies were rated as “unclear” or “high” than as “low.”

### General characteristics of the selected studies

The general characteristics of the eligible studies are summarized in Table 1 in order of publication year. The largest number of published papers originated from the United States (n= 26), followed by Korea (n= 17), and Denmark (n= 5). The average number of firefighters included in each study was 6,103, and there was a significant difference between studies, from a minimum of 86 to a maximum of 45,698. Most studies involved male firefighters (n = 36). Twenty-seven studies involved both male and female. The age groups varied, with most being within the 20-year-old to 60-year-old range. In terms of inter-comparisons, firefighters were compared to the general population, general employees, police officers, military officers, national and regional government officers (NRGs), hospital office workers (HOWs), office workers, and operating engineers. Intra-comparisons examined job type, service years, and rank.

### Twelve categories of non-cancer diseases in firefighters

Given the complexity of the outcome, we categorized non-cancer diseases into 12 categories: (1) infectious and parasitic diseases, (2) endocrine diseases, (3) mental diseases, (4) nervous system diseases, (5) hearing impairment and deafness, (6) circulatory diseases, (7) respiratory diseases, (8) gastrointestinal diseases, (9) musculoskeletal system diseases, (10) genitourinary diseases, (11) suicidal behavior, and (12) other diseases.

We addressed two main outcomes: incidence and mortality. The main outcomes of the eligible studies are summarized in Table 2. Table 2 has also been structured by outcome categories, within which studies are ordered by publication year.

### Infectious and parasitic diseases

Ten studies identified specific infectious and parasitic diseases [16-25]. In inter-comparisons, most studies indicated a risk decrease ranging from 0.16 to 0.42 in terms of infectious diseases in male firefighters compared to the general population [16,21,23,24]. Male firefighters had significantly lower tuberculosis mortality rates than the general population [17], but 1 study did not show significant results [21]. Moreover, firefighters had a higher seroprevalence of hepatitis C antibodies detected in immunoassays than police officers [20].

### Endocrine diseases

Twelve studies regarding endocrine diseases were identified [16,17,19,21,23-30]. Concerning inter-comparisons, most studies indicated a significant decrease (0.10 to 0.67) in the endocrine disease SMR in male firefighters compared to the general population, employees, and military [21,23-25]. Furthermore, most studies reported that the mortality rates of diabetes in male firefighters were lower than those in the general population and employees [16,17,21,23,25,30]. The prevalence of diabetes was lower in male firefighters than in non-firefighters [26]. Furthermore, firefighters had lower risks of diabetes mellitus and type 2 diabetes mellitus than NRGs [29].

### Mental diseases

Eighteen studies that examined mental diseases were identified [19,21,24,25,29,31-43]. Regarding inter-comparisons, male firefighters had significantly higher mortality rates of mental illnesses than the general population in 1 study [19]; compared with NRGs, firefighters had a higher risk of mental illnesses (HR, 1.11; 95% CI, 1.08 to 1.13), mood disorders (HR, 1.12; 95% CI, 1.08 to 1.16), and post-traumatic stress disorder (PTSD; HR, 1.40; 95% CI, 1.26 to 1.56) [29]. The age-standardized prevalence of mental disorders in firefighters was slightly, but not significantly higher, than in government officials and police officers [38]. Deceased firefighters were less likely to have been diagnosed with depression but more likely to have been diagnosed with PTSD than non-firefighters [41].

Concerning job types, emergency medical service (EMS) personnel had a higher rate of PTSD than administration department personnel (OR, 3.68; 95% CI, 1.47 to 9.23) [32]. The risk of PTSD was the highest among paramedics and rescue workers [39]. Retired firefighters reported significantly higher rates of probable PTSD (OR, 2.61; 95% CI, 1.47 to 4.64) and depression (OR, 4.31; 95% CI, 2.27 to 8.22) than current firefighters [35]. Moreover, the risks of severe depression and anxiety disorder were highest among retired firefighters [39]. Professional firefighters have higher excessive daytime sleepiness (EDS) than volunteer firefighters [43]. However, professional firefighters who worked only as firefighters had a significantly lower probability of suffering from PTSD than firefighters who worked temporarily (OR, 0.30; 95% CI, 0.10 to 0.90) [36].

Regarding rank, firefighters at a lower rank were more likely to experience complex PTSD (OR, 1.78; 95% CI, 1.01 to 3.13) [42] and had higher rates of depression [40]; this contrasts with the trend observed for anxiety disorders, where the OR for sergeants was 2.20 (95% CI, 1.06 to 4.57) compared to privates [37]. However, another study reported no significant differences in the prevalence of anxiety disorders according to rank [40].

### Nervous system disease

Thirteen studies have examined nervous system diseases in firefighters [19,21,24,25,29,30,39,43-48]. Regarding inter-comparison, most studies indicated a decrease ranging between 0.47 and 0.68 in nervous system disease risk in male firefighters compared to the general population and employees [21,24,25,44]. Firefighters had a higher risk of sleep disorder (HR, 1.04; 95% CI, 1.01 to 1.08) compared with NRGs [29]. Furthermore, the proportion of firefighters with mild and severe insomnia was higher than HOWs [47].

Concerning the job types, the OR of insomnia was 2.45 (95% CI, 1.46 to 4.12) for fire suppression and 1.87 (95% CI, 1.10 to 3.16) for EMS and rescue personnel [48]. Among the types of duties, the risk of poor sleep quality was the highest among office administrators [39]. Regarding years of service, the ratio of poor sleep significantly decreased with the length of service. It was highest at 52.5% in firefighters with less than 10 years of service, followed by 51.3% in those with 10 years to 20 years of service, and lowest at 37.7% in firefighters with more than 20 years of service [45].

### Hearing impairment and deafness

Seven studies examined hearing impairments and deafness [34,49-54]. Concerning inter-comparison, male firefighters had HL compared with the age-matched otologically normal population [49]. Moreover, male firefighters’ age-adjusted hearing threshold levels were significantly worse than the otologically normal male population (PR, 5.29; 95% CI, 3.34 to 8.39) [53]. Rescuers also had significantly worse hearing than the non-industrial noise-exposed male Korean population [53]. Regarding intra-comparison, the hearing threshold at 4,000 Hz significantly increased with more years spent on the job [51]. Low and high frequency HL prevalence was significantly higher among firefighters with longer work experience than those with shorter work experience [52].

### Circulatory disease

Twenty-two studies that explored circulatory disease [16,17,19,21,23-26,29,30,34,38,44,46,55-62]. Concerning inter-comparison, most studies indicated a 0.27 to 0.86 decrease in circulatory disease in male firefighters compared to the general population [16,21,23,24,55]; however, female firefighters had a significantly higher SMR from circulatory disease [21].

Mortality due to heart disease is significantly lower in male firefighters than in the general population [17], but some studies have reported a lack of association [19,57]. Male firefighters have significantly lower mortality rates from cardiovascular diseases (CVD) than the general population, while female firefighters have significantly higher mortality rates [21]. Most studies reported that the mortality rates of cerebrovascular disease in firefighters was significantly lower than the general population and police officers [19,23,30,44,57,61]. However, firefighters had a significantly higher risk of death or hospitalization from myocardial infarction (MI; HR, 1.24; 95% CI, 1.07 to 1.43) and ischemic stroke (HR, 1.43; 95% CI, 1.12 to 1.82) than the general population [62].

Compared with male employees, firefighters had a higher risk of angina pectoris (SIR, 1.16; 95% CI, 1.08 to 1.24), acute MI (SIR, 1.16; 95% CI, 1.06 to 1.26), chronic ischemic heart disease (IHD; SIR, 1.15; 95% CI, 1.06 to 1.24) and atrial fibrillation/flutter (SIR, 1.25; 95% CI, 1.14 to 1.36) [46]. Concerning the job types, the OR of mortality from coronary heart disease were 12.1 times to 136.0 times higher in fire suppression personnel, 2.8 times to 14.1 times higher in alarm response personnel, and 2.2 times to 10.5 times higher in alarm return personnel as compared with individuals on non-emergency duties [59]. Furthermore, the risk of sudden cardiac death was significantly increased in firefighters responsible for fire suppression, alarm response, and alarm return [60]. Compared to those with less than 10 years of experience as a wildland firefighter, those with 10-19 years of experience had greater odds of having ever been diagnosed with hypertension (OR, 4.2; 95% CI, 1.3 to 14.0), as did those with 20 or more years of experience (OR, 5.0; 95% CI, 1.3 to 20.2) [34].

### Respiratory disease

Sixteen studies examining respiratory disease were reviewed [16,17,19,21,23-25,30,34,44,55,57,58,63-65]. Concerning intercomparison, male firefighters had lower mortality rates from respiratory diseases than the general population [16,17,21,23,24,30,44]. The SMR for pneumonia in male firefighters was significantly lower than that in the general population and employees [21,25]; however, unlike male firefighters, female firefighters had a higher but non-significant SMR for pneumonia than the general population [21].

The SMR for bronchitis, emphysema, and asthma in male firefighters was significantly lower than that of the working population [25]; emphysema deaths in male firefighters were significantly lower than those in the general population [17]. Full-time firefighters had significantly increased asthma rates compared with military personnel (SIR, 1.58; 95% CI, 1.32 to 1.88), while the risk of chronic obstructive pulmonary disease showed no significant results [65].

### Gastrointestinal disease

Eleven studies discussing gastrointestinal disease have been identified [16,17,19,21,23-25,29,30,44,66]. The SMR for liver disease in male firefighters was significantly lower than that in the general population [23]. Furthermore, the SMR for liver and bile duct diseases in male firefighters was significantly lower than that in non-firefighter male employees [25]. However, compared with the general population, significant increases in cirrhosis and chronic liver disease mortality in firefighters have been consistently reported [17,30].

### Musculoskeletal system disease

Thirteen studies about musculoskeletal system disease were reviewed [24,29,34,38,47,67-74]. Concerning inter-comparison, male firefighters had significantly lower SMR for musculoskeletal system diseases than the general population [24]. Male firefighters were reported to have a greater likelihood of experiencing facet joint degeneration (FJD) than HOWs at all lumbar spinal levels except for lumbar (L)3-L4 [71]. Furthermore, the proportions of firefighters with herniated disc(s) (L4-L5) and spinal stenosis (L5- S1) were significantly higher than that of HOWs [47]. The OR of lower back pain (LBP) in EMS and rescue personnel was also statistically higher than that in HOWs [47].

Regarding job types, volunteer firefighters had a 1.7-fold increase in burn-related mortality rate and a 1.8-fold increase in trauma-related mortality than career firefighters [74]. L4-L5 intervertebral disc herniation OR in firefighting, emergency, or rescue personnel was 3.49 (95% CI, 1.24 to 9.86) [70]. EMS and fire suppression workers experienced injuries on duty more often than office workers, and their odds of injuries were three and two times higher, respectively [69].

Regarding years of service, firefighters with more than 17 years of fire services were more likely to report injuries [67]. Furthermore, during the 13-year follow-up period, the prevalence of radiating and local LBP increased [68]. The prevalence of LBP also increases with the duration of years spent in EMS and emergency rescue work [72].

### Genitourinary disease

Seven studies examining genitourinary disease were identified and reviewed [16,19,21,24,25,44,75]. Regarding the inter-comparison, most studies indicated a genitourinary disease SMR decrease between 0.38 and 0.54 in male firefighters as compared to the general population [16,21,44]. The SMR for chronic nephritis in male firefighters is lower than that in the general population [16]. The models assessing male-factor infertility yielded the highest HR among full-time firefighters (HR, 1.46; 95% CI, 1.10 to 1.94) in the male. In Vitro Fertilization model and the male National Patient Register model (HR, 1.53; 95% CI, 1.18 to 1.98), compared to employees [75].

### Suicidal behavior

Twelve studies examined suicidal behavior [16,19,21,23,25,41,44,58,76-79]. Concerning the inter-comparison, firefighters had a higher PMR of 1.72 (95% CI, 1.53 to 1.93) than employees [79]. However, most studies reported that male firefighters have a lower SMR from suicide than the general population [16,19,21,25, 44,58,77], employees, and the military [25]. Mortality due to intentional self-harm was significantly lower among firefighters than among the general population [23]. Intentional self-harm was significantly higher in firefighters employed for over 20 years compared with never-firefighters and firefighters employed for less than 10 years [23]. In contrast, individuals with fewer years of service as firefighters were more likely to report career suicidal ideation, plans, attempts, and non-suicidal self-injury [76]. Furthermore, firefighter decedents had a lower proportion of history of suicide attempts or thoughts than non-firefighter decedents [41].

## DISCUSSION

We conducted a systematic review to identify the risk of noncancer disease in firefighters. A total of 66 studies were identified in our systematic search of the four main databases. During the literature selection process, we excluded studies related to the World Trade Center (WTC) disaster that occurred on September 11, 2001 [82]. The WTC disaster released a large quantity and variety of toxicants into the environment, affecting rescue and recovery workers as well as community members. As cancers, lung disease, heart disease, and PTSD have been shown to be markedly increased, we excluded them.

Most studies have included both male and female firefighters. The risk of death for male and female firefighters was not equal compared with the general population. Male firefighters have significantly lower mortality rates from infectious and parasitic diseases, respiratory disease, pneumonia, gastrointestinal disease, and suicide than the general population; however, unlike male fighters, female firefighters’ mortality rates are not significantly different compared to the general population. Furthermore, the SMR for circulatory disease and CVD in female firefighters was significantly higher than that in general population. However, no study has targeted only female firefighters. Since female firefighters do not account for a large proportion of all firefighters, it is difficult to recruit only female firefighters as subjects in studies, and, as a result, in systematic reviews. Literature about female firefighters and the risk of non-cancerous disease currently lacks evidence. Further studies are needed to understand the risk of non-cancerous diseases in this population.

First, the main results of our study confirmed that the healthy worker effect (HWE) is strong in firefighters as compared with the general population. Firefighters are selected and trained, and so they are expected to be much healthier than the general public. Hence, firefighters are at a lower risk of morbidity and mortality due to disease than the general population. Since most studies had results affected by the HWE, firefighters consistently displayed a lower risk of non-cancer disease than the general population. Therefore, it is essential to match firefighters with an appropriate comparison group to minimize the influence of HWE on statistical analysis and outcomes.

Second, we found that specific non-cancer disease may occur more frequently in firefighters than in other occupational groups, such as police officers, NRGs, military workers, HOWs, and employees. Police officers and military personnel have demographic characteristics similar to firefighters, with comparable socioeconomic status, access to medical services, and physical condition [83]. However, male firefighters had more deaths from respiratory diseases [55], higher hepatitis C [20], and burnout prevalence [66] compared with police officers. Furthermore, the SMR for congenital malformations of the circulatory system in male firefighters and the incidence of asthma in firefighters was higher than military personnel [25,65].

Compared with HOWs, male firefighters have a higher prevalence of insomnia, FJD, spinal stenosis, and disc herniation [47,71]. Furthermore, firefighters responsible for rescue and paramedic services have higher LBP than HOWs [47]. This is estimated to increase the likelihood of musculoskeletal disorders, owing to the necessity for rapid use of force and unnatural posture when transporting patients on stretchers [84]. Compared with general employees, male firefighters have a higher incidence of circulatory diseases, such as angina pectoris, acute MI, chronic IHD, atrial fibrillation, and CVD [46]. Furthermore, full-time male firefighters have a higher risk of infertility than workers as hyperthermia, a major firefighting hazard, may impair male fertility [75,85].

Different studies comparing suicide mortality between firefighter and employees report inconsistent results. Danish firefighters had significantly lower suicide mortality than workers [25], while the United States firefighters had a significantly higher suicide mortality rate than employees [79]. The reason for the disparity between the results seems to be firefighter education on suicide symptoms and prevention, which differs from country to country. Firefighter suicide is a serious problem; from 2011 to 2020, in Korea 97 firefighters attempted suicide; of those, 49 succeeded; approximately twice as many as their deaths [86]. Although the direct cause of firefighter suicide has not been directly linked to the occupation itself, it is estimated that serious mental and physical pain experienced in the field may increase the risk of suicide.

Third, we confirmed that there was a difference in the risk of non-cancer disease depending on job type, years of service, and rank. Concerning job type, firefighters working on fire suppression, alarm response, and alarm return had high rates of sudden cardiac deaths [60] and coronary artery disease deaths [59]. Rescue and paramedic firefighters were shown to be at high risk of PTSD [32,79] as firefighters often encounter serious conditions and witness the death of patients during their duties [87]. Field firefighters had high insomnia [48] and disc herniation incidences [70], while officers were at high-risk of sleep disorders [39] and suicidal thoughts [78]. Moreover, firefighter decedents were more likely to have been diagnosed with PTSD than non-firefighter decedents [41].

Retired firefighters experience more emotional problems, such as depression and anxiety disorders [35,39] and have a higher incidence of PTSD, than non-retired firefighters [35]. People who have experienced retirement are psychologically stressed by the feeling that they no longer fulfill an important role and stress and psychological trauma were accumulated [88]. Seasonal firefighters have a higher incidence of PTSD [36]. This is thought to be due to the increased risk of accidents and mental health issues because of the relative lack of professional training, education on trauma response, and experience that professional firefighter have. In contrast, professional firefighters have a higher prevalence of EDS and sleep disorders [43].

Regarding years of service, the longer firefighters worked, the larger the incidence of deaths from intentional self-harm [23]. Furthermore, the prevalence of hepatitis B [22], HL [51,52], and injuries [67] was higher among those who worked for a long time. We believe that, with longer years of service, capacity for concentration and safety awareness decrease due to aging, which leads to increased accidents. In contrast, the shorter the years of service, the higher the prevalence of sleep disorders [45]. Furthermore, firefighters with fewer years of service were more likely to report suicide attempts, suicidal thoughts, suicide plans, and non-suicide self-harm [76].

Inconsistent results were found for PTSD related to firefighter rank. In a Korean study using a cross-sectional study, firefighters showed significant differences in PTSD incidence according to ranks [32]. However, in a British study using a cohort study, there were no significant differences [42]. Furthermore, inconsistent results have been found for rank-related anxiety disorders. A Brazilian study showed significant differences in the occurrence of anxiety disorders between firefighter ranks [37], while a Chinese study showed no significant differences [40]. This could be due to the different study designs, social/cultural variances, or both; further research is needed.

This is the first systematic review that comprehensively analyzes the risk of various non-cancer disease in firefighters. We used recognized literature search methods to minimize the risk of bias in our methodology. These findings appear to be largely in line with a systematic review of non-cancer occupational health risks in firefighters [13]. Inconclusive findings about increased risk of coronary heart disease and respiratory ill-health in firefighters were reported. They also found that the risk of HL, hip and knee osteoarthritis, mental issues, and sarcoidosis increased in firefighters, with limited evidence. This is consistent with our finding that the evidence of various non-cancer disease is insufficient. Although the risk of specific non-cancer disease in firefighters was increased in this study, the evidence for each disease is commonly cited from only one or two studies. More research for further evidence is required.

This study had several limitations. First, this study was conducted as a descriptive analysis, not a meta-analysis, because of the large heterogeneity of the included studies with respect to the diagnostic criteria, comparison group, and outcome. Differences in the effect size, outcome, and comparison group made it difficult to compare the results of the included studies. Moreover, some were often limited by selection, performance, confirmation, attrition, and reporting biases; which might be interpreted with caution. Second, we limited the publications to those in the English and Korean languages. However, since the majority of scientific literature is published in English, the authors felt that the publication bias caused by limiting selection to only English or Korean was minor.

Based on the results of our study, we make the following suggestions. Firstly, it is necessary to establish and operate a systematic surveillance system for firefighters. The United States has been operating the National Firefighter Registry since 2020 to monitor and track firefighters to analyze the correlation between occupational exposure and cancer occurrence [89]. A surveillance system that includes musculoskeletal system disease, circulatory disease, and mental disease should be established. To understand the trend of diseases occurring in firefighters over time, it is necessary to collect and investigate data systematically and continuously. This will contribute to the prevention of diseases and improvement of firefighter health. Second, HWE should be considered when comparing firefighters’ health issues with those of non-firefighters. Firefighters should be compared with appropriate occupational groups rather than with the general population.

## CONCLUSION

We found that the risk of diabetes, CVD, cerebrovascular disease, acute respiratory infection, pneumonia, liver disease, and intentional self-harm in male firefighters was significantly lower than that in the general population. These results indicate that HWE is an important factor for firefighters. Furthermore, the incidence of lumbar disc herniation, LBP, angina pectoris, acute MI, and PTSD was consistently reported to be significantly higher in firefighters than in occupational groups. We also found that the risk of non-cancer disease differed depending on job type, years of service, and rank. However, caution should be exercised when interpreting the results. It was difficult to generalize the results because the classification criteria for firefighters’ jobs and ranks differ by country. It is also not possible to generalize these results to all firefighters because the included studies were conducted on males or both males and females. Further studies are needed to determine the risk of non-cancer disease among female firefighters.

## Figures and Tables

**Figure f2-epih-44-e2022109:**
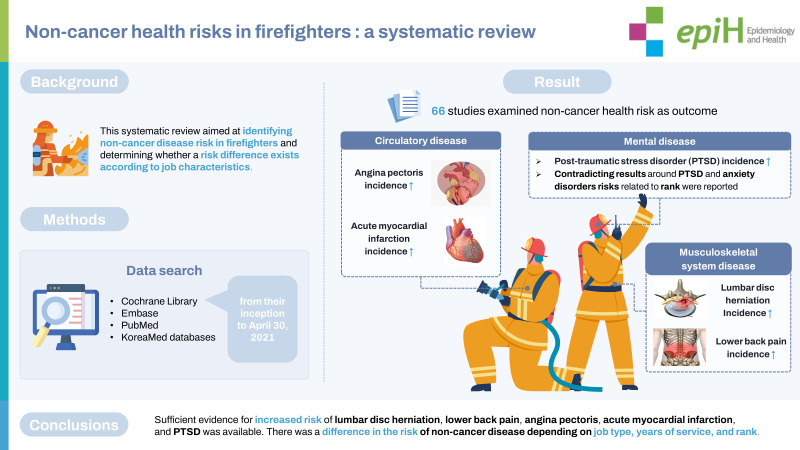


**Table 1.** General characteristics of the included studies (n=66)

Year of publication	RN	First author	Country	No. of firefighters	Sex	Age (mean±SD)	Comparison
1978	16	Musk AW	USA	5,655	M	N.S.	White males in Massachusetts
1989	17	Markowitz JS	USA	86	M	21-57 (37.2±8.8)	Non-exposed firefighters
1990	18	Rosénstock L	USA	4,392	M	≥15	- USA general population
							- Police officers
1990	19	Hansen ES	Denmark	886	M	15-69	Civil servants and employees
1991	20	Beaumont JJ	USA	3,066	M	N.S.	USA general population
1992	21	Demers PA	USA	4,401	M	≥18	Police officers
1993	22	Woodruff BA	USA	510	M	N.S.	American male
1993	23	Guidotti TL	Canada	3,328	M	N.S.	General male population
1995	24	Deschamps S	France	830	M	N.S.	French male population
1999	25	Prezant DJ	USA	11,315	M	≥26	Emergency medical services
2001	26	Baris D	USA	7,789	M	≥16	USA white male population
2001	27	Upfal MJ	USA	678	M, F	20-75	Police officers
2001	28	Kales SN	USA	319	M	≥20	Otologically normal general population
2005	29	Ma F	USA	36,813	M, F	≥20	Florida general population
2005	30	Clark WW	USA	12,609	M	25-64	Non–occupationally exposed groups
2007	31	Kales SN	USA	1,144	N.S.	≥20	Non-emergency duties
2007	32	Miedinger D	Switzerland	101	M	41±11	Swiss study on air pollution and lung diseases in adults Basel population
2008	33	Saijo Y	Japan	1,301	M, F	18-60 (44.1±10.4)	Type of job
2009	34	Ribeiro M	Brazil	1,235	M, F	33±6.1	Police officers
2011	35	Kim MG	Korea	171	M, F	N.S.	Years worked in fire services
2012	36	Hong O	USA	437	M, F	44.9±8.1	Years worked in fire services
2012	37	Mochtar I	Qatar	142	M	30-74 (38.5±5.5)	Non-firefighter employees
2012	38	Shin DY	Korea	1,261	M	≥20 (42.9±7.3)	- Current work department
							- Firefighter rank
2012	39	Contrera-Moreno L	Brazil	308	M, F	36.4±6.5	Years worked in fire services
2013	40	Haddock CK	USA	458	M	38.2±9.9	USA general population
2013	41	Hong O	USA	425	M, F	25-70 (44.8±7.9)	Years worked in fire services
2014	42	Farioli A	USA	206	N.S.	≤45	Non-emergency duties
2014	43	Lim DK	Korea	657	M	N.S.	Years worked in fire services
2015	44	Stanley IH	USA	1,027	M, F	18-82 (38.4±11.7)	- Firefighter rank
							- Years worked in fire services
2015	45	Ahn YS	Korea	29,453	M	20-60 (41.8±9.3)	- Korean male population
							- Non-firefighters and employed <10 yr
2015	46	Kang TS	Korea	912	M	24-59 (44±8)	- Otologically normal male Korean population
							- Non-industrial noise-exposed male Korean population
2015	47	Amadeo B	France	10,829	M	17-64 (30)	French male population
2015	48	Lusa S	Finnish	360	M	35.7±5.4	Years worked in fire services
2016	49	Stanley IH	USA	4,395	M	≥18	USA general male population
2016	50	Semmens EO	USA	499	M, F	40±13	Years worked in wildland firefighting
2016	51	Hong O	USA	154	M, F	45.3±7.9	Operating engineers
2016	52	Yoon JH	Korea	19,119	M	20-60	Type of job
2016	53	Jang TW	Korea	392	M, F	20-59	Administrative firefighters
2016	54	Strauß M	Germany	97	M	23-58 (40.5±9)	Office workers
2016	55	Harvey SB	Australia	753	M, F	≥18	Retired firefighters
2017	56	Kim DH	Korea	341	M	20-59	Hospital office workers
2017	57	Lee W	Korea	257	M	30-59	Korean male workers
2017	58	Kim MG	Korea	24,209	M	20-59	Duration of each job (yr)
2018	59	Phelps SM	USA	249	M, F	41.8±8.3	Primary position
2018	60	Han M	Korea	23,356	M, F	39.5±9.0	National and regional government officer
2018	61	Muegge CM	India	2,818	M, F	≥18	General population
2018	62	Psarros C	Greece	102	M	40.0±8.2	Seasonally professional firefighters
2018	63	Pedersen JE_1	Denmark	11,691	M	10-60 (52±14.5)	Male employees
2018	64	Pedersen JE_2	Denmark	11,968	M	10-60 (52±14.5)	Military employees
2018	65	Petersen KU	Denmark	11,775	M	N.S	- Male employees
							- Military employees
2019	66	Kahn S	USA	3,159	N.S.	N.S.	Type of job
2019	67	Lin PY	Taiwan	9,328	M	≥20	Policemen
2019	68	Park H	Korea	45,698	M, F	42.5±9.1	Type of job
2019	69	Kim MG	Korea	297	M	20-59 (38.9±9.8)	Hospital office workers
2019	70	Petersen KU	Denmark	4,710	M	N.S	- Male employees
							- Military employees
2019	71	Azevedo DSS	Brazil	711	M	19-50	Firefighter rank
2020	72	Pinkerton L	USA	29,992	M, F	≥17	USA general population
2020	73	Noh J	Korea	8,242	M, F	≥40 (45.9±4.8)	General population
2020	74	Jang TW	Korea	9,738	M, F	N.S.	Type of job
2020	75	Min J	Korea	31,743	M, F	20-60	- Police officers
							- Government workers
2020	76	Kim YT	Korea	1,022	M, F	41.7±10.5	Type of job
2020	77	Chen X	China	335	M, F	18-51 (27.3±6.1)	Firefighter rank
2021	78	Vigil NH	USA	298	M, F	18-90	USA working population
2021	79	Pennington ML	USA	722	M, F	47.2±17.4	Non-firefighters
2021	80	Langtry J	UK	1,300	M, F	18-61 (47.4±8.8)	Firefighter rank
2021	81	Savall A	France	193	M, F	39.1±11.5	Volunteer firefighters

RN, reference number; M, male; F, female; N.S., not specified; SD, standard deviation.

**Table 2.** Outcomes of the 12 disease categories related to firefighters

RN		Sex	Comparison^1^	Outcomes related to mortality (95% CI)	Outcomes related to incidence and prevalence (95% CI)	
(1) Infectious and parasitic diseases						
	[16]	M	[1]	SMR 0.42		
	[23]	M	[1]	SMR 0.33 (0.04, 1.20)		
	[29]	M, F	[1]	Male FF: SMR 0.16 (0.11, 0.22)^*^		
				Female FF: SMR 0.27 (0.00, 1.52)		
	[45]	M	[1]	SMR 0.30 (0.14, 0.55)^*^		
	[47]	M	[1]	SMR 0.30 (0.16, 0.50)^*^		
	[65]	M	[2], [3]	[2] SMR 0.76 (0.44, 1.31)		
				[3] SMR 1.10 (0.64, 1.90)		
	Tuberculosis					
	[20]	M	[1]	RR 0.26 (0.07, 0.68)^2*^		
	[29]	M	[1]	SMR 1.10 (0.35, 2.56)		
	Hepatitis B virus infection					
	[22]	M	[1]		FF 4.5%, [1] 6.8%	
	[39]	M, F	Length of service, rank		≥20 yr 19.6%, 10-20 yr 4.2%, <10 yr 3.3%^*^	
					Rank was not associated with a higher prevalence of hepatitis B infection	
	Hepatitis C virus infection					
	[27]	M, F	[4]		FF 2.3%, [4] 0.6%	
(2) Endocrine diseases						
	[29]	M	[1]	SMR 0.35 (0.22, 0.52)^*^		
	[45]	M	[1]	SMR 0.44 (0.25, 0.72)^*^		
	[47]	M	[1]	SMR 0.67 (0.44, 0.98)^*^		
	[65]	M	[2], [3]	[2] SMR 0.10 (0.01, 0.73)^*^		
				[3] SMR 0.12 (0.02, 0.87)^*^		
	Diabetes mellitus					
	[16]	M	[1]	SMR 0.39		
	[20]	M	[1]	RR 0.36 (0.14, 0.75)^2*^		
	[29]	M	[1]	SMR 0.45 (0.26, 0.73)^*^		
	[37]	M	[2]		FF 6.3%^*^, [2] 14.1%^*^	
	[45]	M	[1]	SMR 0.45 (0.25, 0.75)^*^		
	[60]	M, F	[5]		HR 0.73 (0.69, 0.77)^*^	
	[65]	M	[2], [3]	[2] SMR 0.58 (0.37, 0.89)^*^		
				[3] SMR 0.80 (0.51, 1.24)		
	[72]	M, F	[1]	SMR 0.73 (0.64, 0.83)^*^		
	Type 2 diabetes mellitus					
	[60]	M, F	[5]		HR 0.85 (0.82, 0.88)^*^	
	Endocrine disorders					
	[23]	M	[1]	SMR 0.46 (0.09, 1.37)		
	Metabolic syndrome					
	[54]	M	[6]		FF 14.4%^*^, [6] 32.6%^*^	
					Clerical: OR 1.15 (0.69, 1.92)	
					Managerial: OR 1.52 (0.79, 2.89)	
					Professionals and related: OR 1.20 (0.75, 1.94)	
					Service: OR 0.63 (0.27, 1.45)	
	[57]	M	[FF]		Sales: OR 2.34 (1.29, 4.23)^*^	
					Skilled agricultural, fishing: OR 0.84 (0.40, 1.74)	
					Craft and related trades: OR 0.97 (0.55, 1.72)	
					Equipment and assembling: OR 1.27 (0.76, 2.12)	
					Elementary: OR 1.10 (0.53, 2.28)	
(3) Mental diseases						
	[23]	M	[1]	SMR 4.55 (2.74, 7.11)^*^		
	[29]	M	[1]	SMR 0.41 (0.22, 0.68)^*^		
	[47]	M	[1]	SMR 0.57 (0.38, 0.81)		
	[60]	M, F	[5]		HR 1.11 (1.08, 1.13)^*^	
	[65]	M	[2], [3]	[2] SMR 0.37 (0.25, 0.57)^*^		
				[3] SMR 0.47 (0.31, 0.71)^*^		
	Depression					
	[33]	M, F	[Other shift work]		Firefighting: OR 1.17 (0.60, 2.30)	
					Ambulance: OR 1.55 (0.76, 3.14)	
					Rescue: OR 1.01 (0.46, 2.20)	
	[50]	M, F	[<10 yr]		10-19 yr: OR 1.7 (0.6, 4.4)	
					≥20 yr: OR 0.8 (0.2, 3.4)	
	[55]	M, F	[Current FF]		Retired FF: unadjusted OR 4.31 (2.27, 8.22)^*^	
	[76]	M, F	Type of duty		School educator: 1.00%, fire-control: 1.79%, paramedics and rescue: 1.88%, office administrator: 1.62%, retirees: 3.95%	
	Mood disorder					
	[60]	M, F	[5]		HR 1.12 (1.08, 1.16)^*^	
	[75]	M, F	[4], [5]		No significant difference in age-adjusted prevalence between the 3 job categories	
	Anxiety disorder					
	[71]	M	[Privates]		Corporal: OR 1.51 (0.70, 3.28)	
					Sergeant: OR 2.20 (1.06, 4.57)^*^	
					Sub-lieutenant: OR 0.85 (0.10, 7.09)	
					Officer: OR 1.59 (0.41, 6.17)	
	[75]	M, F	[4], [5]		No significant difference in age-adjusted prevalence between the 3 job categories	
	[76]	M, F	Type of duty		School educator: 0.00%, fire-control: 0.67%, paramedics and rescue: 0.47%, office administrator: 1.08%, retirees: 2.63%	
	[77]	M, F	FF rank		Cadet/FF: 10.6%, engineer/driver/captain: 2.7%, officer, chief of staff: 7.2%	
	Post-traumatic stress disorder					
	[38]	M	[FF], [Administration]		[FF]	
						Senior fire sergeant: OR 1.28 (0.72, 2.28)
						Fire sergeant: OR 2.28 (1.28, 4.06)^*^
						Fire lieutenant: OR 2.47 (1.14, 5.37)^*^
						Fire captain: OR 2.88 (1.11, 7.45)^*^
						Fire chief: OR 2.11 (0.48, 9.28)
					[Administration]	
						Firefighting: OR 2.04 (1.00, 4.17)
						Rescue: OR 2.22 (0.84, 5.86)
						EMS: OR 3.68 (1.47, 9.23)^*^
	[55]	M, F	[Current FF]		Retired FF: unadjusted OR 2.61 (1.47, 4.64)^*^	
	[60]	M, F	[5]		HR 1.40 (1.26, 1.56)^*^	
	[62]	M	[Temporary FF]		Permanent FF: OR 0.30 (0.10, 0.90)^*^	
	[76]	M, F	Type of duty		School educator: 6.00%, fire-control: 10.94%, paramedics and rescue: 14.08%, office administrator: 9.19%, retirees: 11.84%	
	[79]	M, F	[2]		FF 4.56%^*^, [2] 1.73%^*^	
	[80]	M, F	[Group manager or higher]		FF/control operator: OR 1.50 (0.51, 4.40)	
					Crew manager: OR 1.75 (0.61, 4.99)	
					Watch manager: OR 1.69 (0.61, 4.65)	
					Station manager: OR 2.41 (0.87, 6.70)	
	Complex post-traumatic stress disorder					
	[80]	M, F	[Group manager or higher]		FF/control operator: OR 1.78 (1.01, 3.13)^*^	
					Crew manager: OR 1.45 (0.82, 2.57)	
					Watch manager: OR 1.08 (0.62, 1.89)	
					Station manager: OR 1.04 (0.56, 1.92)	
	Stress disorder					
	[75]	M, F	[4], [5]		No significant difference in age-adjusted prevalence between the 3 job categories	
	Excessive daytime sleepiness					
	[40]	M	Type of duty		On duty 13.7%, off-duty 14.0%	
	[81]	M, F	Volunteer FF		Professional FF: 38.2%^*^, volunteer FF: 23.5%^*^	
	Non-organic sleep disorders					
	[75]	M, F	[4], [5]		No significant difference in age-adjusted prevalence between the 3 job categories	
(4) Nervous system diseases						
	[23]	M	[1]	SMR 0.73 (0.20, 1.88)		
	[26]	M	[1]	SMR 0.47 (0.27, 0.83)^*^		
	[29]	M	[1]	SMR 0.54 (0.31, 0.86)^*^		
	[47]	M	[1]	SMR 0.68 (0.46, 0.96)^*^		
	[65]	M	[2], [3]	[2] SMR 0.50 (0.32, 0.78)^*^		
				[3] SMR 0.76 (0.48, 1.19)		
	[72]	M, F	[1]	SMR 0.89 (0.80, 1.00)		
	Obstructive sleep apnea					
	[81]	M, F	Volunteer FF		Professional FF: 3.6%, volunteer FF: 0.7%	
	Sleep disorders					
	[43]	M	Tenure (yr)		≥20 yr: 37.7%^*^, 10-20 yr: 51.3%^*^, <10 yr: 52.5%^*^	
	[60]	M, F	[5]		HR 1.04 (1.01, 1.08)^*^	
	[76]	M, F	Type of duty		School educator: 50.00%, fire-control: 53.79%, paramedics and rescue: 53.05%, office administrator: 56.22%, retirees: 36.84%	
	[81]	M, F	Volunteer FF		Professional FF: 37.5%^*^, volunteer FF: 22.6%^*^	
	Insomnia					
	[69]	M	[7]		FF 14.5%^*^, [7] 1.3%^*^	
	[74]	M, F	[Administrative]		Fire suppression: OR 2.45 (1.46, 4.12)^*^	
					EMS/rescue: OR 1.87 (1.10, 3.16)^*^	
	[81]	M, F	Volunteer FF		Professional FF: 19.9%, volunteer FF: 16.4%	
	Transient ischemic attack					
	[63]	M	[2]		SIR 1.12 (0.97, 1.30)	
(5) Hearing impairment and deafness						
	Hearing loss					
	[28]	M	[1]		HF hearing loss: RR 2.9 (1.7, 5.1)^*^	
					Broad frequency hearing loss: RR 2.9 (1.5, 5.6)^*^	
	[30]	M	[8]		FF did not exhibit excessive loss of hearing compared with the comparison groups	
	[35]	M, F	Work period		4,000 Hz mean (SD)	
					<5 yr: 9.6 (6.2)^*^, 5-9 yr: 12.0 (9.5)^*^, 10-14 yr: 15.1 (9.6)^*^, ≥15 yr: 27.9 (16.2)^*^	
	[41]	M, F	Yr in fire services		HF hearing loss: <5 yr: 5.0%^*^, 5-9 yr: 25.5%^*^, 10-19 yr: 32.2%^*^, 20-29 yr: 52.7%^*^, ≥30 yr: 74.4%^*^	
					LF hearing loss: <5 yr: 0.0%^*^, 5-9 yr: 0.0%^*^, 10-19 yr: 3.8%^*^, 20-29 yr: 10.9%^*^, ≥30 yr: 15.4%^*^	
	[46]	M	[9], [10]		[9] PR 5.29 (3.34, 8.39)^*^, [10] PR 0.99 (0.95, 1.03)	
					[10] Rescuer: PR 1.005 (1.002, 1.007)^*^	
					Paramedic: PR 1.001 (0.998, 1.005)	
					Suppressor: PR 0.999 (0.997, 1.000)	
					Office worker: PR 0.999 (0.997, 1.001)	
	[50]	M, F	[<10 yr]		10-19 yr: OR 1.7 (0.6, 4.4)	
					≥20 yr: OR 2.0 (0.6, 6.0)	
	[51]	M, F	[11]		HF hearing loss: FF 34.4%, [11] 58.7%	
					LF hearing loss: FF 3.9%, [11] 19.9%	
	Tinnitus					
	[51]	M, F	[10]		FF 40.3%, [11] 39.4%	
(6) Circulatory diseases						
	[16]	M	[1]	SMR 0.86		
	[18]	M	[1], [4]	[1] SMR 0.81 (0.73, 0.89)^*^		
				[4] SMR 0.83		
				[4] IDR 0.91 (0.76, 1.09)		
	[29]	M, F	[1]	Male FF: SMR 0.69 (0.63, 0.76)^*^		
				Female FF: SMR 2.49 (1.32, 4.25)^*^		
	[45]	M	[1], [non-FF and employed <10 yr]	[1] SMR 0.27 (0.20, 0.37)^*^		
				[non-FF and employed <10 yr]		
				10-20 yr: ARR 1.57 (0.72, 3.41)		
				≥20 yr: ARR 1.40 (0.72, 2.74)		
	[47]	M	[1]	SMR 0.76 (0.68, 0.85)^*^		
	Heart disease					
	[20]	M	[1]	RR 0.89 (0.81, 0.97)^2*^		
	[21]	M	[4]	IDR 0.86 (0.74, 1.00)		
	[23]	M	[1]	SMR 1.10 (0.92, 1.31)		
	Rheumatic heart disease					
	[16]	M	[1]	SMR 0.29		
	Hypertension					
	[37]	M, F	[2]		FF 26.7%, [2] 34.8%	
	[50]	M, F	[<10 yr]		10-19 yr: OR 4.2 (1.3, 14.0)^*^	
					≥20 yr: OR 5.0 (1.3, 20.2)^*^	
	[60]	M	[5]		HR 0.85 (0.82, 0.88)^*^	
	[65]	M, F	[2], [3]	[2] SMR 0.96 (0.54, 1.69)		
				[3] SMR 1.01 (0.57, 1.77)		
	[75]	M, F	[4], [5]		No significant difference in age-adjusted prevalence between the 3 job categories	
	Angina pectoris					
	[60]	M	[5]		HR 1.06 (1.02, 1.10)^*^	
	[63]	M, F	[2]		SIR 1.16 (1.08, 1.24)^*^	
	Myocardial infarction					
	[73]	M, F	[1]	HR 1.24 (1.07, 1.43)^*^		
	Acute myocardial infarction					
	[60]	M	[5]		HR 1.21 (1.10, 1.32)^*^	
	[63]	M	[2]		SIR 1.16 (1.06, 1.26)^*^	
	Ischemic heart disease					
	[19]	M	[2]	SMR 1.15 (0.74, 1.71)		
	[20]	M	[1]	RR 0.95 (0.87, 1.04)^2^		
	[21]	M	[4]	IDR 0.88 (0.74, 1.04)		
	[23]	M	[1]	SMR 1.05 (0.86, 1.27)		
	[24]	M	[1]	SMR 0.74 (0.20, 1.90)		
	[26]	M	[1]	SMR 1.09 (1.02, 1.16)^*^		
	[45]	M	[1], [non-FF and employed <10 yr]	[1] SMR 0.42 (0.25, 0.66)^*^		
				[non-FF and employed <10 yr]		
				10-20 yr: ARR 1.35 (0.44, 4.14)		
				≥20 yr: ARR 0.93 (0.34, 2.51)		
	[61]	M, F	[1]	OR 0.98 (0.88, 1.10)		
	[65]	M	[2], [3]	[2] SMR 0.86 (0.73, 1.02)		
				[3] SMR 1.00 (0.84, 1.18)		
	[72]	M, F	[1]	SMR 0.98 (0.95, 1.01)		
	[75]	M, F	[4], [5]		No significant difference in age-adjusted prevalence between the 3 job categories	
	Chronic ischemic heart disease					
	[63]	M	[2]		SIR 1.15 (1.06, 1.24)^*^	
	Heart failure					
	[63]	M	[2]		SIR 1.01 (0.91, 1.12)	
	Sudden cardiac death					
	[42]	N.S.	[Non-emergency duties]	Fire suppression: RR 22.1 (14.8, 32.9)^*^		
				Alarm response: RR 2.6 (1.5, 4.6)^*^		
				Alarm return: RR 4.1 (2.7, 6.2)^*^		
	Atrial fibrillation/flutter					
	[63]	M	[2]		SIR 1.25 (1.14, 1.36)^*^	
	CHD					
	[31]	N.S.	[Non-emergency duties]	Fire suppression: RR 12.1-136.0^*^		
				Alarm response: RR 2.8-14.1^*^		
				Alarm return RR: 2.2-10.5^*^		
	[37]	M	[2]		FF CHD risk score 6.5% (SD 3.7)^*^	
					[2] CHD risk score 9.5% (SD 6.5)^*^	
	Cardiovascular diseases					
	[29]	M, F	[1]	Male FF: SMR 0.73 (0.65, 0.83)^*^		
				Female FF: SMR 3.85 (1.66, 7.58)^*^		
	[63]	M	[2]		SIR 1.10 (1.05, 1.15)^*^	
	Cerebrovascular diseases					
	[20]	M	[1]	RR 0.84 (0.67, 1.03)^2^		
	[21]	M	[4]	IDR 0.65 (0.45, 0.92)^*^		
	[23]	M	[1]	SMR 0.38 (0.17, 0.73)^*^		
	[24]	M	[1]	SMR 1.16 (0.24, 3.38)		
	[26]	M	[1]	SMR 0.83 (0.69, 0.99)^*^		
	[45]	M	[1], [non-FF and employed <10 yr]	[1] SMR 0.24 (0.14, 0.38)^*^		
				[non-FF and employed <10 yr]		
				10-20 yr: ARR 1.30 (0.31, 5.43)		
				≥20 yr: ARR 1.87 (0.63, 5.59)		
	[60]	M, F	[5]		HR 0.97 (0.90 1.04)	
	[61]	M, F	[1]	OR 0.82 (0.67, 0.99)^*^		
	[65]	M	[2], [3]	[2] SMR 0.81 (0.61, 1.07)		
				[3] SMR 0.91 (0.69, 1.21)		
	[72]	M, F	[1]	SMR 0.90 (0.83, 0.97)^*^		
	[75]	M, F	[4], [5]		No significant difference in age-adjusted prevalence between the 3 job categories	
	Ischemic stroke					
	[73]	M, F	[1]	HR 1.43 (1.12, 1.82)^*^		
	Hemorrhagic stroke					
	[73]	M, F	[1]	HR 1.13 (0.74, 1.73)		
	Arteriosclerosis					
	[23]	M	[1]	SMR 1.49 (0.77, 2.61)		
	[63]	M	[2]		SIR 1.02 (0.88, 1.18)	
(7) Respiratory diseases						
	[16]	M	[1]	SMR 0.93		
	[18]	M	[1], [4]	[1] SMR 0.88 (0.66, 1.17)		
				[4] SMR 1.41		
				[4] unadjusted IDR 1.98 (1.09, 3.87)^*^		
	[20]	M	[1]	RR 0.63 (0.40, 0.95)^2*^		
	[21]	M	[4]	IDR 1.11 (0.71, 1.73)		
	[26]	M	[1]	SMR 0.67 (0.55, 0.82)^*^		
	[29]	M, F	[1]	Male FF: SMR 0.50 (0.35, 0.70)^*^		
				Female FF: SMR 2.88 (0.58, 8.43)		
	[45]	M	[1], [non-FF and employed <10 yr]	[1] SMR 0.13 (0.03, 0.37)^*^		
				[non-FF and employed <10 yr]		
				≥20 yr: ARR 5.89 (0.34, 101.13)		
	[47]	M	[1]	SMR 0.54 (0.39, 0.73)^*^		
	[72]	M, F	[1]	SMR 0.81 (0.76, 0.86)^*^		
	Acute respiratory infection					
	[20]	M	[1]	RR 0.63 (0.40, 0.95)^2*^		
	[65]	M	[2], [3]	[2] SMR 1.15 (0.16, 8.14)		
				[3] SMR 1.60 (0.23, 11.35)		
	Pneumonia					
	[21]	M	[4]	IDR 1.04 (0.46, 2.36)		
	[29]	M, F	[1]	Male FF: SMR 0.34 (0.17, 0.62)^*^		
				Female FF: SMR 4.35 (0.49, 15.70)		
	[65]	M	[2] [3]	[2] SMR 0.36 (0.16, 0.80)^*^		
				[3] SMR 0.48 (0.22, 1.07)		
	Bronchitis, emphysema and asthma					
	[65]	M	[2], [3]	[2] SMR 0.71 (0.52, 0.98)^*^		
				[3] SMR 0.94 (0.69, 1.30)		
	Asthma					
	[17]	M	Non-exposed FF		FF 9.4%, non-exposed FF 0.0%	
	[20]	M	[1]	RR 0.31 (0.01, 1.75)^2^		
	[34]	M, F	[4]		FF 8.7%, [4] 7.2%	
	[50]	M, F	[<10 yr]		10-19 yr: OR 1.4 (0.5, 3.5)	
					≥20 yr: OR 2.0 (0.5, 8.3)	
	[64]	M	[3]		SIR 1.58 (1.32, 1.88)^*^	
	Bronchitis					
	[17]	M	Non-exposed FF		FF 14.3%, non-exposed FF 0.0%	
	Chronic bronchitis					
	[20]	M	[1]	RR 0.74 (0.15, 2.17)^2^		
	[24]	M	[1]	SMR 1.83 (0.05, 10.21)		
	Emphysema					
	[20]	M	[1]	RR 0.52 (0.24, 0.99)^2*^		
	[21]	M	[4]	IDR 1.45 (0.54, 3.88)		
	[26]	M	[1]	SMR 0.64 (0.40, 1.02)		
	[29]	M, F	[1]	Male FF: SMR 0.87 (0.40, 1.66)		
				Female FF: SMR 12.5 (0.16, 69.60)		
	Chronic obstructive pulmonary disease					
	[20]	M	[1]	RR 0.75 (0.43, 1.23)^2^		
	[21]	M	[4]	IDR 1.11 (0.65, 1.89)		
	[23]	M	[1]	SMR 1.57 (0.78, 2.81)		
	[64]	M	[3]		SIR 1.14 (0.98, 1.32)	
	[72]	M, F	[1]	SMR 0.78 (0.71, 0.85)^*^		
(8) Digestive diseases						
	[16]	M	[1]	SMR 0.85		
	[20]	M	[1]	RR 1.57 (1.27, 1.92)^2*^		
	[23]	M	[1]	SMR 0.46 (0.21, 0.89)^*^		
	[26]	M	[1]	SMR 0.97 (0.81, 1.17)		
	[29]	M, F	[1]	Male FF: SMR 0.57 (0.43, 0.73)^*^		
				Female FF: SMR 1.60 (0.18, 5.78)		
	[45]	M	[1], [non-FF and employed <10 yr]	[1] SMR 0.24 (0.16, 0.34)		
				[non-FF and employed <10 yr]		
				≥20 yr: ARR 1.17 (0.40, 3.44)		
	[47]	M	[1]	SMR 0.79 (0.65, 0.95)^*^		
	Diseases of the stomach and duodenum					
	[20]	M	[1]	RR 1.57 (0.93, 2.49)^2^		
	Diseases of the oral cavity, esophagus and stomach					
	[65]	M	[2], [3]	[2] SMR 0.43 (0.16, 1.15)		
	Gastric and duodenal ulcers			[3] SMR 0.60 (0.22, 1.59)		
	[26]	M	[1]	SMR 0.67 (0.38, 1.17)		
	Peptic ulcer disease					
	[60]	M, F	[5]		HR 1.13 (1.11, 1.15)^*^	
	[67]	M	[4]		Self-reported PUD: FF 7.1%^*^, [4] 8.3%^*^	
					Self-reports of physician-diagnosed PUD: FF 5.5%^*^, [4] 6.5%^*^	
	Hernia and intestinal obstruction					
	[20]	M	[1]	RR 0.64 (0.13, 1.89)^2^		
	Liver diseases					
	[45]	M	[1], [non-FF and employed <10 yr]	[1] SMR 0.26 (0.17, 0.37)^*^		
				[non-FF and employed <10 yr]		
				≥20 yr: ARR 1.42 (0.46, 4.38)		
	Alcoholic liver disease					
	[60]	M, F	[5]		HR 0.80 (0.76, 0.83)^*^	
	Cirrhosis and chronic liver disease					
	[20]	M	[1]	RR 2.27 (1.73, 2.93)^2*^		
	[26]	M	[1]	SMR 1.10 (0.84, 1.43)		
	[72]	M, F	[1]	SMR 1.16 (1.03, 1.29)		
	Diseases of the liver and bile duct					
	[65]	M	[2], [3]	[2] SMR 0.73 (0.54, 0.98)^*^		
				[3] SMR 0.85 (0.63, 1.15)		
(9) Musculoskeletal system diseases						
	[47]	M	[1]	SMR 0.00 (0.00, 0.58)^*^		
	[75]	M, F	[4], [5]		No significant difference in age-adjusted prevalence between the 3 job categories	
	Non-rheumatoid arthritis					
	[50]	M, F	[<10 yr]		10-19 yr: OR 2.0 (0.7, 6.4)	
					≥20 yr: OR 1.5 (0.4, 5.8)	
	Facet joint degeneration					
	[56]	M	[7]		L1-L2: OR 2.64 (1.31, 5.31)^*^	
					L2-L3: OR 2.28 (1.30, 4.00)^*^	
					L3-L4: OR 1.41 (0.81, 2.46)	
					L4-L5: OR 1.91 (1.03, 3.54)^*^	
					L5-S1: OR 1.81 (1.03, 3.18)^*^	
	Spinal stenosis					
	[69]	M	[7]		FF 33.0%, [7] 20.8%^*^	
	Lumbar disc herniation					
	[53]	M, F	[Administrative]		L1-L2 intervertebral disc: OR 1.19 (0.44, 3.22)	
					L2-L3 intervertebral disc: OR 1.33 (0.50, 3.55)	
					L3-L4 intervertebral disc: OR 1.73 (0.48, 6.22)	
					L4-L5 intervertebral disc: OR 3.49 (1.24, 9.86)^*^	
					L5-S1 intervertebral disc: OR 1.18 (0.47, 2.96)	
	[60]	M, F	[5]		HR 1.43 (1.39, 1.46)^*^	
	[69]	M	[7]		FF 51.9%^*^, [7] 36.4%^*^	
	LBP					
	[48]	M	Yr in fire services		During the 13 yr follow-up, the prevalence of radiating LBP increased from 16 to 29% and that of local LBP from 28 to 40%^*^	
	[58]	M	Duration of each job (yr)		LBP increased according to the increment of duration of EMS and ER work^*^	
	[60]	M, F	[5]		HR 1.52 (1.43, 1.63)^*^	
	[69]	M	[7]		EMS: OR 2.57 (1.20, 5.58)^*^	
					Rescue: OR 3.69 (1.37, 9.94)^*^	
	Job-related injuries					
	[36]	M, F	[<17 yr]		≥17 yr: OR 2.96 (1.92, 4.58)^*^	
	[52]	M	[Officer]		Fire suppression: OR 1.86 (1.61, 2.15)^*^	
					EMS: OR 2.93 (2.51, 3.42)^*^	
	[59]	M, F	Primary position		Primary position was not significantly associated with work related injury	
	[66]	N.S	[Career FF]	Volunteer burns: OR 1.7 (1.2, 2.4)^*^		
				Volunteer trauma: OR 1.8 (1.5, 2.2)^*^		
(10) Genitourinary diseases						
	[16]	M	[1]	SMR 0.49		
	[23]	M	[1]	SMR 1.32 (0.57, 2.60)		
	[26]	M	[1]	SMR 0.54 (0.36, 0.81)^*^		
	[29]	M	[1]	SMR 0.38 (0.14, 0.83)^*^		
	[47]	M	[1]	SMR 0.94 (0.50, 1.61)		
	Chronic nephritis					
	[16]	M	[1]	SMR 0.38		
	Nephritis and kidney stones					
	[65]	M	[2], [3]	[2] SMR 0.67 (0.10, 4.79)		
				[3] SMR 1.06 (0.15, 7.56)		
	Male infertility					
	[70]	M	[2], [3]		Male In Vitro Fertilization model: [2] HR 1.46 (1.10, 1.94)^*^, [3] HR 1.40 (1.06, 1.86)^*^	
					Male National Patient Register model: [2] HR 1.53 (1.18, 1.98)^*^, [3] HR 1.36 (1.05, 1.75)^*^	
(11) Suicidal behavior						
	Suicide					
	[16]	M	[1]	SMR 0.19		
	[23]	M	[1]	SMR 0.38 (0.15, 0.79)^*^		
	[24]	M	[1]	SMR 0.47 (0.06, 1.69)		
	[26]	M	[1]	SMR 0.66 (0.48, 0.92)^*^		
	[29]	M, F	[1]	Male FF: SMR 0.55 (0.44, 0.68)^*^		
				Female FF: SMR 2.52 (0.68, 6.44)		
	[49]	M	[1]	FF 11.61 per 100,000 PY, [1] 18.53 per 100,000 PY		
	[65]	M	[2], [3]	[2] SMR 0.65 (0.48, 0.87)^*^		
				[3] SMR 0.78 (0.57, 1.05)		
	[78]	M, F	[2]	PMR 1.72 (1.53, 1.93)^*^		
	[44]	M, F	[Lower rank], length of service		[Lower rank]	
					Higher rank: OR 1.04 (0.72, 1.51)	
					Officer rank: OR 0.14 (0.06, 0.35)^*^	
					FF with fewer yr of service were more likely to report suicide attempts (b=-0.14)^*^	
	Suicide attempt					
	[79]	M, F	[2]		FF 24.21%^*^, [2] 33.39%^*^	
	Suicidal ideation					
	[44]	M, F	[Lower rank], length of service		[Lower rank]	
					Higher rank: OR 0.97 (0.73, 1.30)	
					Officer rank: OR 0.64 (0.44, 0.92)^*^	
					FF with fewer yr of service were more likely to report career suicidal ideation (b=-0.03)^*^	
	[68]	M, F	[Fire suppression]		EMS: OR 0.76 (0.70, 0.82)^*^	
					Officers: OR 1.48 (1.36, 1.62)^*^	
	Suicide plan					
	[44]	M, F	[Lower rank], length of service		[Lower rank]	
						Higher rank: OR 0.77 (0.54, 1.10)
						Officer rank: OR 0.33 (0.19, 0.59)^*^
					FF with fewer yr of service were more likely to report suicide plans (b=-0.08)^*^	
	Non-suicidal self-injury					
	[44]	M, F	[Lower rank], length of service		[Lower rank]	
						Higher rank: OR 0.93 (0.65, 1.34)
						Officer rank: OR 0.10 (0.03, 0.27)^*^
					FF with fewer yr of service were more likely to report non-suicidal self-injury (b=-0.15)^*^	
	Intentional self-harm					
	[45]	M, F	[1], [non-FF and employed <10 yr]	[1] SMR 0.45 (0.33, 0.60)^*^		
				[non-FF and employed <10 yr]		
				10-20 yr: ARR 0.56 (0.28, 1.12)		
				≥20 yr: ARR 2.57 (1.01, 6.64)^*^		
(12) Other diseases						
	Circulatory congenital malformations					
	[65]	M	[2], [3]	[2] SMR 2.35 (0.76, 7.28)		
				[3] SMR 6.95 (2.24, 21.54)^*^		
	Sarcoidosis					
	[25]	M	EMS Prehospital health-care workers		FF	
						Annual incidence proportions: 12.9/100,000
						Point prevalence: 222/100,000
					EMS prehospital health-care workers	
						Annual incidence proportions: 0
						Point prevalence: 35/100,000
	Atopy					
	[32]	M	[1], duration of employment		FF 51%^*^, [1] 32%^*^	
					Duration of employment did not account for the differences in prevalence of atopy	
	Burnout					
	[67]	M	[4]		FF 24.2%^*^, [4] 22.5%^*^	

In this study, the criterion for the SMR, IDR, or PMR was 1; In studies that reported results by multiplying the SMR, IDR, or PMR by 100, the corresponding result was divided by 100 and organized in a table. RN, reference number; M, male; F, female; N.S., not specified; CI, confidence interval; RR, relative risk; ARR, adjusted relative risk; SMR, standardized mortality ratio; SIR, standardized incidence ratio; HR, hazard ratio; OR, odds ratio; PR, prevalence ratio; IDR, incidence density ratio; PMR, proportionate mortality ratio; EMS, emergency medical service; ER, emergency rescue; FF, firefighters; HF, high frequency; LF, low frequency; PY, person years; LBP, lower back pain; SD, standard deviation; PUD, peptic ulcer disease; CHD, coronary heart disease.

1 The numbers and words in [ ] of the comparison indicate the reference category of the study; The comparison groups are denoted as follows: [1], general population; [2], employees; [3], military employees; [4], police officers; [5], national and regional government officer; [6], office workers; [7], hospital office workers; [8], non–occupationally exposed groups of individuals; [9], otologically normal male Korean population; [10], non-industrial noise-exposed male Korean population.

2 In this study, rate ratio (RR): where 1.00 indicates an equal number of observed and expected events.

* p<0.05.

## SUPPLEMENTARY MATERIALS

Supplementary Material 1. Searching strategy in PubMed, KoreaMed, EMBASE and Cochrane Central Register of Controlled Trials (CENTRAL)

Supplementary Material 2 Diagnostic criteria of the non-cancer diseases related to firefighters

Supplementary Material 3 Risk of bias graph of included studies
