# Supplementary material for: Non-cancer health risks in firefighters: a systematic review
Source: Epidemiol Health. 2022 Nov 16;44:e2022109. doi: 10.4178/epih.e2022109 (PMC10396521; doi:10.4178/epih.e2022109)
Supplement: Supplementary Material 1. — Searching strategy in PubMed, KoreaMed, EMBASE and Cochrane Central Register of Controlled Trials (CENTRAL) [file epih-44-e2022109-Supplementary-1.docx]

**Supplementary Material 1.** Searching strategy in PubMed, KoreaMed, EMBASE and Cochrane Central Register of Controlled Trials (CENTRAL)

| Database type | Searching strategy |
| --- | --- |
| PubMed | #1 (firefighter[MeSH Terms]) OR (firefighting)  #2 ((((((disease [MeSH Terms]) OR (incidence) OR (prevalence) OR (mortality) OR (injury) OR (death) OR (occupational relevance)  #3 #1 AND #2  #4 #1 AND #2, Filters: English, Korean |
| KoreaMed | firefighter |
| EMBASE | #1 firefighter OR firefighting  #2 disease OR incidence OR prevalence OR mortality OR injury OR death OR 'occupational relevance’  #3 #1 AND #2  #4 #1 AND #2 AND ([english]/lim OR [korean]/lim) |
| Cochrane  Central  Register of  Controlled  Trials  (CENTRAL) | #1 MeSH descriptor: [Firefighters] explode all trees  #2 firefighting  #3 #1 OR #2  #4 MeSH descriptor: [Disease] explode all trees  #5 incidence  #6 prevalence  #7 mortality  #8 injury  #9 death  #10 occupational relevance  #11 #4 OR #5 OR #6 OR #7 OR #8 OR #9 OR #10  #12 #3 AND #11 |
