# Supplementary material for: Non-cancer health risks in firefighters: a systematic review
Source: Epidemiol Health. 2022 Nov 16;44:e2022109. doi: 10.4178/epih.e2022109 (PMC10396521; doi:10.4178/epih.e2022109)
Supplement: Supplementary Material 2 — Diagnostic criteria of the non-cancer diseases related to firefighters [file epih-44-e2022109-Supplementary-2.docx]

**Supplementary Material 2.** Diagnostic criteria of the non-cancer diseases related to firefighters

| Disease | First author (year) | Diagnostic criteria | Disease definition (ICD code) |
| --- | --- | --- | --- |
| 1) Infectious and parasitic diseases | | | |
| Infectious and parasitic diseases | Musk AW (1978) | ICD-7 | Infective (001-139) |
|  | Guidotti TL (1993) | ICD-9 | Infectious, parasitic disorders (001-139) |
|  | Ma F (2005) | ICD-9 | Infectious diseases |
|  | Ahn YS (2015) | ICD-10 | Infection (A00-B99) |
|  | Amadeo B (2015) | ICD | Infectious and parasitic diseases |
|  | Petersen KU (2018) | ICD-8 and ICD-10 | Infections (A00-09, A20-99, B00-89, B91-97, B99) |
| Tuberculosis | Beaumont JJ (1991) | ICD | Tuberculosis |
|  | Ma F (2005) | ICD-9 | Tuberculosis |
| Hepatitis B virus infection | Woodruff BA (1993) | Radioimmunoassay test | Hepatitis B virus infection |
|  | Contrera-Moreno L (2012) | ELISA | Hepatitis B virus infection |
| Hepatitis C virus infection | Upfal MJ (2001) | ELISA | Hepatitis C virus infection |
| 2) Endocrine disease | | | |
| Endocrine, nutritional and metabolic disease | Ma F (2005) | ICD-9 | Allergic/endocrine |
|  | Ahn YS (2015) | ICD-10 | Endocrine diseases (E00-E90) |
|  | Amadeo B (2015) | ICD | Endocrine, nutritional and metabolic diseases |
|  | Petersen KU (2018) | ICD-8 and ICD-10 | Endocrine and nutritional disorders (E00-07, E15-90) |
| Diabetes mellitus | Musk AW (1978) | ICD-7 | Diabetes (260) |
|  | Beaumont JJ (1991) | ICD | Diabetes mellitus |
|  | Ma F (2005) | ICD-9 | Diabetes |
|  | Mochtar I (2012) | physician-diagnosed | Diabetes mellitus |
|  | Ahn YS (2015) | ICD-10 | Diabetes (E10-E14) |
|  | Han M (2018) | ICD-10 | Diabetes mellitus |
|  | Petersen KU (2018) | ICD-8 and ICD-10 | Diabetes (E10-14) |
|  | Pinkerton L (2020) | ICD-10 | Diabetes mellitus |
| Type 2 diabetes mellitus | Han M (2018) | ICD-10 | Type 2 diabetes mellitus |
| Endocrine disorder | Guidotti TL (1993) | ICD-9 | Endocrine disorders (240-279) |
| Metabolic syndrome | Strauß M (2016) | Criteria of the IDF in 2005 | Metabolic syndrome |
|  |  |  | continued |
| Disease | First author (year) | Diagnostic criteria | Disease definition (ICD code) |
| Metabolic syndrome | Lee W (2017) | US National Cholesterol Education Program Adult Treatment Panel III criteria | Metabolic syndrome |
| 3) Mental disease | | | |
| Mental disease | Guidotti TL (1993) | ICD-9 | Mental disorder (290-319) |
|  | Ma F (2005) | ICD-9 | Mental |
|  | Amadeo B (2015) | ICD | Mental and behavioral disorders |
|  | Petersen KU (2018) | ICD-8 and ICD-10 | Mental disorders (F00-99) |
|  | Han M (2018) | ICD-10 | Mental illnesses |
| Depression | Saijo Y (2008) | CES-D ≥ 16 | Depressive symptom |
|  | Semmens EO (2016) | Questionnaire | Depression |
|  | Harvey SB (2016) | DSM-IV Criterion A | Depression |
|  | Kim YT (2020) | CES-D ≥ 23 | Depression |
|  | Chen X (2020) | SDS ≥ 50 | Depressive disorders |
|  | Pennington ML (2021) | ICD-10 | depression/dysthymia or serious mental illness |
| Mood disorder | Han M (2018) | ICD-10 | Mood disorder |
|  | Min J (2020) | ICD-10 | Mood disorder (F30–F39) |
| Anxiety disorder | Azevedo DSS (2019) | physician-diagnosed | Anxiety disorder |
|  | Chen X (2020) | SAS ≥ 50 | Anxiety |
|  | Min J (2020) | ICD-10 | Anxiety disorder (F41) |
|  | Kim YT (2020) | BAI ≥ 32 | Anxiety disorder |
| Post-traumatic  stress disorder (PTSD) | Shin DY (2012) | IES ≥ 26 | Posttraumatic stress disorder |
|  | Harvey SB (2016) | DSM-IV Criterion A | Post-traumatic stress disorder |
|  | Han M (2018) | ICD-10 | Traumatic stress disorder |
|  | Psarros C (2018) | ICD-10 | Post-traumatic stress disorder |
|  | Kim YT (2020) | PCL-S < 11 | Post-traumatic stress disorder |
|  | Pennington ML (2021) | ICD-10 | Posttraumatic stress disorder |
|  | Langtry J (2021) | ITQ in accordance with ICD-11 | Posttraumatic stress disorder |
| Complex  post-traumatic stress disorder | Langtry J (2021) | ITQ in accordance with ICD-11 | Complex post-traumatic stress disorder |
| Stress disorder | Min J (2020) | ICD-10 | Stress disorder (F43) |
| Excessive daytime sleepiness | Haddock CK (2013) | ESS > 10 | Excessive daytime sleepiness |
|  |  |  | continued |
| Disease | First author (year) | Diagnostic criteria | Disease definition (ICD code) |
| Excessive daytime sleepiness | Savall A (2021) | ESS ≥ 11 | Excessive daytime sleepiness |
| Non-organic sleep disorder | Min J (2020) | ICD-10 | Nonorganic sleep disorders (F51) |
| 4) Nervous system disease | | | |
| Nervous system disease | Guidotti TL (1993) | ICD-9 | Nervous system disorders (320-389) |
|  | Baris D (2001) | ICD-9 | Nervous system diseases (320-389) |
|  | Ma F (2005) | ICD-9 | Nervous System |
|  | Amadeo B (2015) | ICD | Diseases of the nervous system and the sense organs |
|  | Petersen KU (2018) | ICD-8 and ICD-10 | Nervous system and sensory organs (G04-99, H00-95) |
|  | Pinkerton L (2020) | ICD-10 | Nervous system disorders |
| Obstructive sleep apnea | Savall A (2021) | STOP-BANG ≥ 5 | Obstructive sleep apnea |
| Sleep disorder | Lim DK (2014) | PSQI ≥ 6 | Sleep disorder |
|  | Han M (2018) | ICD-10 | Sleep disorders |
|  | Kim YT (2020) | PSQI ≥ 6 | Sleep disorder |
|  | Savall A (2021) | PSQI ≥ 6 | Sleep disorder |
| Insomnia | Kim MG (2019) | ISI-K ≥ 15 | Insomnia |
|  | Jang TW(2020) | ISI ≥ 15 | Insomnia |
|  | Savall A (2021) | ISI ≥ 15 | Insomnia |
| Transient ischaemic attack | Pedersen JE_1 (2018) | ICD-8 and ICD-10 | Transient ischaemic attack (435.09– 435.99, G45) |
| 5) Hearing impairment and deafness | | | |
| Hearing loss | Kales SN (2001) | -High frequency hearing loss: HTL greater than 30 dB at 3000, 4000 and 6000 Hz  -Broad frequency hearing loss: HTL greater than 20 dB at 500, 1000, 2000, and 4000 Hz | -High frequency hearing loss  -Broad frequency hearing loss |
|  | Clark WW (2005) | Audiometric test | occupational noise–induced hearing loss |
|  | Kim MG (2011) | Hearing test | occupational noise–induced hearing loss |
|  | Hong O (2013) | Audiometric test | Hearing loss |
|  | Kang TS (2015) | pure-tone average (PTA) of the HTLs for both ears that exceeds 25 dB at 1000, 2000, 3000 and 4000 Hz (PTA1234) | Hearing loss |
|  | Semmens EO (2016) | Questionnaire | Hearing loss |
|  | Hong O (2016) | HTL of 25 dB or greater in the worst ear | Hearing loss |
| Tinnitus | Hong O (2016) | Questionnaire | Tinnitus |
|  |  |  | continued |
| Disease | First author (year) | Diagnostic criteria | Disease definition (ICD code) |
| 6) Circulatory disease | | | |
| Circulatory disease | Musk AW (1978) | ICD-7 | Circulatory (400-468) |
|  | Rosénstock L (1990) | ICD | Non-malignant circulatory diseases |
|  | Ma F (2005) | ICD-9 | Circulatory system |
|  | Ahn YS (2015) | ICD-10 | Circulatory diseases (I00-I99) |
|  | Amadeo B (2015) | ICD | Diseases of the circulatory system |
| Heart disease | Beaumont JJ (1991) | ICD | Disease of the heart |
|  | Demers PA (1992) | ICD-9 | Heart disease (390-398, 402, 404, 410-414, 420-429) |
|  | Guidotti TL (1993) | ICD-9 | Heart (390-98, 402, 404, 410-416, 420-429) |
| Rheumatic heart disease | Musk AW (1978) | ICD-7 | Rheumatic heart disease (410) |
| Hypertension | Mochtar I (2012) | JNC-7 criteria | Hypertension |
|  | Semmens EO (2016) | Questionnaire | Hypertension |
|  | Han M (2018) | ICD-10 | Hypertension |
|  | Petersen KU (2018) | ICD-8 and ICD-10 | Hypertension (I10-15) |
|  | Min J (2020) | ICD-10 | Hypertensive disease (I10–I15) |
| Angina pectoris | Han M (2018) | ICD-10 | Angina pectoris |
|  | Pedersen JE_1 (2018) | ICD-8 and ICD-10 | Angina pectoris (413, I20) |
| Myocardial infarction | Noh J (2020) | KCD | Myocardial infarction |
| Acute myocardial infarction | Han M (2018) | ICD-10 | Acute myocardial infarction |
|  | Pedersen JE_1 (2018) | ICD-8 and ICD-10 | Acute myocardial infarction (410, 411, I21) |
| Ischemic heart disease (IHD) | Hansen ES (1990) | ICD-8 | Ischaemic heart disease (410-414) |
|  | Beaumont JJ (1991) | ICD | Ischemic heart disease |
|  | Demers PA (1992) | ICD-9 | Ischaemic heart disease (410-414) |
|  | Guidotti TL (1993) | ICD-9 | Ischemia heart disease (410-414) |
|  | Deschamps S (1995) | ICD-9 | Ischaemic heart disease |
|  | Baris D (2001) | ICD-9 | Ischemic heart disease (410-414) |
|  | Ahn YS (2015) | ICD-10 | Ischemic heart diseases (I20-I25) |
|  | Muegge CM (2018) | ICD-9 and ICD-10 | Ischemic heart disease |
|  | Petersen KU (2018) | ICD-8 and ICD-10 | Ischemic heart diseases (I20-25) |
|  | Pinkerton L (2020) | ICD-10 | Ischaemic heart disease (I20- 22, I24- 25, I51.3, I51.6) |
|  | Min J (2020) | ICD-10 | Ischemic heart disease (I20–I25) |
|  |  |  | continued |
| Disease | First author (year) | Diagnostic criteria | Disease definition (ICD code) |
| Chronic IHD | Pedersen JE_1 (2018) | ICD-8 and ICD-10 | Chronic ischemic heart disease (412, I25) |
| Heart failure | Pedersen JE_1 (2018) | ICD-8 and ICD-10 | Heart failure (427.09–11, 427.19, 519.19, I50, J81, J68, R570) |
| Sudden cardiac death (SCD) | Farioli A (2014) | SCD within 24 hours of last fire service duty or sudden cardiac event within 24 hours of last duty followed by permanent loss of consciousness until death | Sudden cardiac death |
| Atrial fibrillation/flutter | Pedersen JE_1 (2018) | ICD-8 and ICD-10 | Atrial fibrillation/flutter (427.93–94, I48) |
| Coronary heart disease | Kales SN (2007) | Narrative reports | Coronary heart disease |
|  | Mochtar I (2012) | Framingham risk score | Coronary heart disease |
| Cardiovascular diseases | Ma F (2005) | ICD-9 | Cardiovascular |
|  | Pedersen JE_1 (2018) | ICD-8 and ICD-10 | Cardiovascular diseases |
| Cerebrovascular disease | Beaumont JJ (1991) | ICD | Cerebrovascular disease |
|  | Demers PA (1992) | ICD-9 | Cerebrovascular disease (430-438) |
|  | Guidotti TL (1993) | ICD-9 | Cerebrovascular disease (430-438) |
|  | Deschamps S (1995) | ICD-9 | Cerebrovascular disease (430-438) |
|  | Baris D (2001) | ICD-9 | Cerebrovascular diseases (430-438) |
|  | Ahn YS (2015) | ICD-10 | Cerebrovascular diseases (I60-I69) |
|  | Han M (2018) | ICD-10 | Cerebrovascular diseases |
|  | Muegge CM (2018) | ICD-9 and ICD-10 | Cerebrovascular disease |
|  | Petersen KU (2018) | ICD-8 and ICD-10 | Cerebrovascular diseases (I60-69) |
|  | Pinkerton L (2020) | ICD-10 | Cerebrovascular disease (G45.0-45.2, G45.4-45.9, I60- I69) |
|  | Min J (2020) | ICD-10 | Cerebrovascular disease (I60–I69) |
| Ischemic stroke | Noh J (2020) | KCD | Ischemic stroke |
| Hemorrhagic stroke | Noh J (2020) | KCD | Hemorrhagic stroke |
| Arteriosclerosis | Guidotti TL (1993) | ICD-9 | Arteriosclerosis (440-448) |
|  | Pedersen JE_1 (2018) | ICD-8 and ICD-10 | Arteriosclerosis (440, I70) |
| 7) Respiratory disease | | | |
| Respiratory disease | Musk AW (1978) | ICD-7 | Respiratory (470-527) |
|  | Rosénstock L (1990) | ICD | Non-malignant respiratory diseases |
|  | Beaumont JJ (1991) | ICD | Disease of the respiratory system |
|  | Demers PA (1992) | ICD-9 | Respiratory disease (460-466, 470-478, 480-487, 490-519) |
|  |  |  | continued |
| Disease | First author (year) | Diagnostic criteria | Disease definition (ICD code) |
| Respiratory disease | Baris D (2001) | ICD-9 | Respiratory disease (460-519) |
|  | Ma F (2005) | ICD-9 | Respiratory diseases |
|  | Ahn YS (2015) | ICD-10 | Respiratory diseases (J00-J99) |
|  | Amadeo B (2015) | ICD | Diseases of the respiratory system |
|  | Pinkerton L (2020) | ICD-10 | Non-malignant respiratory diseases |
| Acute upper respiratory infection | Beaumont JJ (1991) | ICD | Acute respiratory infections |
|  | Petersen KU (2018) | ICD-8 and ICD-10 | Acute airway infections (J00-06, J10-11) |
| Pneumonia | Demers PA (1992) | ICD-9 | Pneumonia (480-486) |
|  | Ma F (2005) | ICD-9 | Pneumonia |
|  | Petersen KU (2018) | ICD-8 and ICD-10 | Pneumonia (J12-18) |
| Brochitis, emphysema and asthma | Petersen KU (2018) | ICD-8 and ICD-10 | Bronchitis, emphysema and asthma (J20-22, J40-47) |
| Asthma | Markowitz JS (1989) | physician-diagnosed | Asthma |
|  | Beaumont JJ (1991) | ICD | Asthma |
|  | *Ribeiro* M (2009) | European Community Respiratory Health Survey (ECRHS) definition | Asthma |
|  | Semmens EO (2016) | Questionnaire | Asthma |
|  | Pedersen JE_2 (2018) | ICD-8 and ICD-10 | Asthma (49300–49309, J45-46) |
| Bronchitis | Markowitz JS (1989) | physician-diagnosed | Bronchitis |
| Chronic bronchitis | Beaumont JJ (1991) | ICD | Chronic and unspecified bronchitis |
|  | Deschamps S (1995) | ICD-9 | Chronic bronchitis (490-492, 494, 496) |
| Emphysema | Beaumont JJ (1991) | ICD | Emphysema |
|  | Demers PA (1992) | ICD-9 | Emphysema (492) |
|  | Baris D (2001) | ICD-9 | Emphysema (492) |
| Chronic obstructive pulmonary disease (COPD) | Beaumont JJ (1991) | ICD | Chronic obstructive and other pulmonary disorder |
|  | Demers PA (1992) | ICD-9 | COPD and other respiratory disease (470-478, 494-519) |
|  | Guidotti TL (1993) | ICD-9 | COPD (490-496) |
|  | Pedersen JE_2 (2018) | ICD-8 and ICD-10 | COPD (49000, 49002–49209, J40-J44.9) |
|  | Pinkerton L (2020) | ICD-10 | COPD (J40- J44) |
| 8) Digestive disease | | | |
| Digestive disease | Musk AW (1978) | ICD-7 | Gastrointestinal (530-587) |
|  | Beaumont JJ (1991) | ICD | Diseases of the digestive system |
|  |  |  | continued |
| Disease | First author (year) | Diagnostic criteria | Disease definition (ICD code) |
| Digestive disease | Guidotti TL (1993) | ICD-9 | Digestive system disorder (520-579) |
|  | Baris D (2001) | ICD-9 | Digestive diseases (520-577) |
|  | Ma F (2005) | ICD-9 | Digestive diseases |
|  | Ahn YS (2015) | ICD-10 | Digestive diseases (K00-K93) |
|  | Amadeo B (2015) | ICD | Diseases of the digestive system |
| Disease of stomach and duodenum | Beaumont JJ (1991) | ICD | Disease of stomach and duodenum |
| Disease of oral cavity, oesophagus and stomach | Petersen KU (2018) | ICD-8 and ICD-10 | Oral cavity, oesophagus and stomach (K00-31) |
| Gastric and duodenal ulcer | Baris D (2001) | ICD-9 | Gastric and duodenal ulcer (531-553) |
| Peptic ulcer disease | Han M (2018) | ICD-10 | Peptic ulcer |
|  | Lin PY (2019) | Self-reported and  self-reports of physician-diagnosed | Peptic ulcer disease |
| Hernia and intestinal obstruction | Beaumont JJ (1991) | ICD | Hernia and intestinal obstruction |
| Liver disease | Ahn YS (2015) | ICD-10 | Liver diseases (K70-K77) |
| Alcoholic liver disease | Han M (2018) | ICD-10 | Alcoholic liver disease |
| Cirrhosis and chronic liver disease | Beaumont JJ (1991) | ICD | Cirrhosis and other liver disease |
|  | Baris D (2001) | ICD-9 | Cirrhosis of liver (571) |
|  | Pinkerton L (2020) | ICD-10 | Cirrhosis and other chronic liver disease (K70, K73-74, K76.0) |
| Disease of liver and bile duct | Petersen KU (2018) | ICD-8 and ICD-10 | Liver and bile ducts (K70-83) |
| 9) Musculoskeletal system disease | | | |
| Musculoskeletal system disease | Amadeo B (2015) | ICD | Diseases of the muscular system/connective tissue |
|  | Min J (2020) | ICD-10 | Musculoskeletal disease (M23, M50, M51) |
| Non-rheumatoid arthritis | Semmens EO (2016) | Questionnaire | Non-rheumatoid arthritis |
| Facet joint degeneration | Kim DH (2017) | Classification method of Pathria et al | Facet joint degeneration |
| Spinal stenosis | Kim MG (2019) | MRI scan | Spinal stenosis |
| Lumbar disc herniation | Jang TW (2016) | questionnaire, MRI, and Pfirrmann methods | Lumbar intervertebral disc degeneration |
|  | Han M (2018) | ICD-10 | Lumbar disc herniation |
|  | Kim MG (2019) | MRI scan | Herniated disc |
| Lower back pain | Lusa S (2015) | Nordic questionnaire | Lower back pain |
|  | Kim MG (2017) | NIOSH I criteria | Lower back pain |
|  | Han M (2018) | ICD-10 | Lower back pain |
|  |  |  | continued |
| Disease | First author (year) | Diagnostic criteria | Disease definition (ICD code) |
| Lower back pain | Kim MG (2019) | KOSHA musculoskeletal questionnaire | Lower back pain |
| Job-related injuries | Hong O (2012) | Questionnaire | Job-related injuries |
|  | Yoon JH (2016) | Structured questionnaires | Job-related injuries |
|  | Phelps SM (2018) | Questionnaire (OSHA 2001 standards) | work-related injury |
|  | Kahn S (2019) | U.S. Fire Administration information | burns, trauma |
| 10) Genitourinary disease | | | |
| Genitourinary system disease | Musk AW (1978) | ICD-7 | Genitourinary (590-639) |
|  | Guidotti TL (1993) | ICD-9 | Genitourinary system disorders (580-629) |
|  | Baris D (2001) | ICD-9 | Genitourinary diseases (580-629) |
|  | Ma F (2005) | ICD-9 | Genitourinary |
|  | Amadeo B (2015) | ICD | Diseases of the genitourinary system |
| Chronic nephritis | Musk AW (1978) | ICD-7 | Chronic nephritis (592) |
| Nephritis and kidney stones | Petersen KU (2018) | ICD-8 and ICD-10 | Nephritis and kidney stones (N00-08, N20-23) |
| Male infertility | Petersen KU (2019) | ICD-8 and ICD-10 | Male infertility |
| 11) Suicide and suicidal behavior | | | |
| Suicide | Musk AW (1978) | ICD-7 | Suicide (979) |
|  | Guidotti TL (1993) | ICD-9 | Suicide (950-959) |
|  | Deschamps S (1995) | ICD-9 | Suicide (E950-E958) |
|  | Baris D (2001) | ICD-9 | Suicide (950-959) |
|  | Ma F (2005) | ICD-9 | Suicide |
|  | Stanley IH (2016) | ICD-10 | Suicide (X60-84) |
|  | Petersen KU (2018) | ICD-8 and ICD-10 | Suicide (X60-84, Y87.0) |
|  | Vigil NH (2021) | ICD–10 | Suicide |
| Suicide attempt | Stanley IH (2015) | modified version of the SITBI-SF | Suicide attempts |
|  | Pennington ML (2021) | ICD-10 | history of suicide attempts or thoughts |
| Suicide ideation | Stanley IH (2015) | modified version of the SITBI-SF | Suicide ideation |
|  | Park H (2019) | SBQ-R ≥ 2 | Suicide ideation |
| Suicide plan | Stanley IH (2015) | modified version of the SITBI-SF | Suicide plans |
| Non-suicidal self-injury | Stanley IH (2015) | modified version of the SITBI-SF | Non-suicidal self-injury |
| Intentional self-harm | Ahn YS (2015) | ICD-10 | Intentional self-harm (X60-X84) |
|  |  |  | continued |
|  |  |  |  |
| Disease | First author (year) | Diagnostic criteria | Disease definition (ICD code) |
| 12) Other diseases | | | |
| Circulatory congenital malformations | Petersen KU (2018) | ICD-8 and ICD-10 | Circulatory congenital malformations (Q20-28) |
| Sarcoidosis | Prezant DJ (1999) | Biopsy | Sarcoidosis |
| Atopy | Miedinger D (2007) | Skin-prick test | Atopy |
| Burnout | Lin PY (2019) | Occupational burnout tool | Burnout |
| ^1^ ICD: International Classification of Diseases, KCD: Korean Standard Classification of Diseases, ELISA: enzyme-linked immunoassay test, CES-D: Center for Epidemiologic Studies Depression Scale, SDS: Self-Rating Depression Scale, DSM: Diagnostic and Statistical Manual of Mental Disorders, BDI-PC: Beck Depression Inventory for Primary Care, SAS: Self-Rating Anxiety Scale, BAI: Beck Anxiety Inventory, IES: Impact of Event Scale, PCL-S: PTSD Checklist-Specific, ITQ: International Trauma Questionnaire, ESS: Epworth Sleepiness Scale, PSQI: Pittsburgh Sleep Quality Index, ISI: Insomnia Severity Index, JNC-7: Joint National Committee on Prevention, Detection, Evaluation, and Treatment of High Blood Pressure, KOSHA: Korea occupational Safety and Health Agency, SITBI-SF: Self-Injurious Thoughts and Behaviors Interview-Short Form, SBQ-R: Suicidal Behavior Questionnaire-Revised | | | |
