# Supplementary figures and images for: Non-cancer health risks in firefighters: a systematic review
Source: Epidemiol Health. 2022 Nov 16;44:e2022109. doi: 10.4178/epih.e2022109 (PMC10396521; doi:10.4178/epih.e2022109)

**Supplementary Material 3.** Risk of bias graph of included studies

**
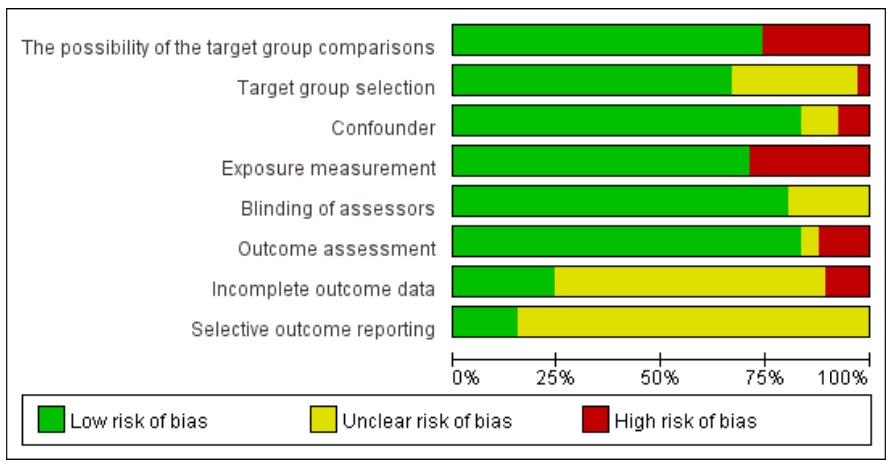
**

Supplement: Supplementary Material 3 — Risk of bias graph of included studies [file epih-44-e2022109-Supplementary-3.docx]
